# Supplementary material for: Increased mental stress among undergraduate medical students in south-western Saudi Arabia during the COVID-19 pandemic
Source: PeerJ. 2022 Aug 15;10:e13900. doi: 10.7717/peerj.13900 (PMC9387517; doi:10.7717/peerj.13900)
Supplement: Supplemental Information 1 [file peerj-10-13900-s001.docx]

**Pre-COVID-19 pandemic questionnaire**

***Dear student:***

This questionnaire is for the purpose of identifying the extent of perceived stress and its associated factors among medical college students. Kindly answer clearly and objectively, and not write anything that indicates your personality or your name in this questionnaire. Thank you very much.

***1- Personal data:***

- Age: …………….(years)

- Gender: Male / Female

- Academic level: ......................................................

- Grade Point Average (GPA):………………………………………..

-Family income: Sufficient and exceed/ Sufficient/ Insufficient

- Smoking status: Smoker/ Ex-smoker/ Non-smoker

***2- Perceived Stress Scale (PSS)***

The questions in this scale ask about your feelings and thoughts during the last month. In each case, you will be asked to indicate how often you felt or thought a certain way. Although some of the questions are similar, there are differences between them and you should treat each one as a separate question. The best approach is to answer fairly quickly. That is, don’t try to count up the number of times you felt a particular way; rather indicate the alternative that seems like a reasonable estimate.

| Question | Never | Almost never | Some-times | Fairly often | Very often |
| --- | --- | --- | --- | --- | --- |
| l. In the last month, how often have you been upset because of something that happened unexpectedly? |  |  |  |  |  |
| 2. In the last month, how often have you felt that you were unable to control the important things in your life? |  |  |  |  |  |
| 3. In the last month, how often have you felt nervous and stressed? |  |  |  |  |  |
| 4. In the last month, how often have you felt confident about your ability to handle your personal problems? |  |  |  |  |  |
| 5. In the last month, how often have you felt that things were going your way? |  |  |  |  |  |
| 6. In the last month, how often have you found that you could not cope with all the things that you had to do? |  |  |  |  |  |
| 7. In the last month, how often have you been able to control irritations in your life? |  |  |  |  |  |
| 8. In the last month, how often have you felt that you were on top of things? |  |  |  |  |  |
| 9. In the last month, how often have you been angered because of things that  happened that were outside of your control? |  |  |  |  |  |
| 10. In the last month, how often have you felt difficulties were piling up so high that  you could not overcome them? |  |  |  |  |  |
