# Supplementary material for: Increased mental stress among undergraduate medical students in south-western Saudi Arabia during the COVID-19 pandemic
Source: PeerJ. 2022 Aug 15;10:e13900. doi: 10.7717/peerj.13900 (PMC9387517; doi:10.7717/peerj.13900)
Supplement: Supplemental Information 2 [file peerj-10-13900-s002.pdf]

استبيان عن الشعور بالارهاق بين طالبات الكليات الصحية بجامعة الملك خالد

عزيزتى الطالبة:

هذا الاستبيان بغرض التعرف على مدى انتشار مرض الارتجاع المعدي و اثاره الصحية بين طالبات الكليات الصحية. و الغرض منه بالاضافة الى تدريب طلاب طب المجتمع في البحوث الصحية هو غرض بحثي بحت. برجاء التكرم بالاجابة بوضوح وموضوعية و عدم كتابة ما يدل على شخصيتك او اسمك في هذا الاستبيان. شاكرين مقدرين.

طالبات المستوى الثامن كلية الطب بمقرر طب المجتمع.

1. البياناتالشخصية:

A-عمر: (سنة) B-الجنس: ○ذكر 1 ○انثى 2

C- الكلية:○الطب 1 ○العلوم الطبية التطبيقية2 ○طب الاسنان 3

○الصيدلة 4 ○ التمريض 5

D-المستوى:○ الثالث 3 ○ الرابع 4 ○ الخامس 5 ○ السادس 6 ○ السابع 7

○ الثامن 8 ○ التاسع 9 ○ العاشر 10 ○ الحادي عشر 11 ○ الثاني عشر 12

E- المعدل التراكمي في السنة السابقة ( )

F- الحالة الاجتماعية○: متزوج 1 ○ أعزب 2

G- دخل الأسرة: ○كافي و يفيض1 ○ كافي بالكاد 2 ○لا يكفي 3

H- التدخين○ :مدخن1 ○ مدخن سابق 2 ○غير المدخن 3

الأسئلة في هذا الاستبيان تتعلق بأحاسيسك وأفكارك خلال الشهر **الماضي**. ويطلب منك في كل سؤال أن تبين كم مرة أحسست أو فكرت بطريقة معينة. وإن كانت بعض الأسئلة متشابهة، غير أن هناك اختلافات بينها، لذلك المرجو منك أن تتعامل مع كل سؤال على أساس أنه سؤال مستقل. والطريقة المثلى هي أن تجيب على كل سؤال بسرعة، أي أن لا تحاول أن تحسب بالضبط عدد المرات التي أحسست بشيء معين، بل أن تجيب على السؤال بتقدير معقول. للإجابة على كل سؤال من الأسئلة التالية، اختر اجابة واحدة

	السؤال	لم يحدث أبدا 0	تقريبا لم يحدث أبدا 1	أحيانا 2	في كثير من الأحيان ولكن الى حد ما 3	كثيرا جدا 4
O	خلال الشهر الماضي كم من الوقت شعرتِ بالاضطراب نتيجة حصول حادثة غير متوقعة؟					
P	خلال الشهر الماضي كم من الوقت شعرتِ بعدم قدرتك على التحكم بالأمور المهمة في حياتك؟					
Q	خلال الشهر الماضي كم من الوقت شعرتِ بالتوتر و"الضغط النفسي"؟					
R	خلال الشهر الماضي كم من الوقت شعرتِ بالثقة حيال قدرتك على التعامل مع مشاكلك الشخصية؟					
S	خلال الشهر الماضي كم من الوقت شعرتِ أن الأمور تجري حسب مشيئتك؟					
T	خلال الشهر الماضي كم من الوقت شعرتِ بعدم قدرتك على التكيف مع جميع الأمور التي عليك فعلها؟					
U	خلال الشهر الماضي كم من الوقت شعرتِ انك قادرة على التحكم بمصادر الازعاج في حياتك؟					
V	خلال الشهر الماضي كم من الوقت شعرتِ انك تتحكم بجميع الأمور؟					
W	خلال الشهر الماضي كم من الوقت شعرتِ بالغضب بسبب أمور خارجة عن سيطرتك؟					
X	خلال الشهر الماضي رخلال الشهر الماضي كم من الوقت شعرتِ ان المصاعب تتراكم لدرجة لا يمكنك التغلب عليها؟					
