# Supplementary material for: Increased mental stress among undergraduate medical students in south-western Saudi Arabia during the COVID-19 pandemic
Source: PeerJ. 2022 Aug 15;10:e13900. doi: 10.7717/peerj.13900 (PMC9387517; doi:10.7717/peerj.13900)
Supplement: Supplemental Information 3 [file peerj-10-13900-s003.docx]

**Pre-COVID-19 pandemic questionnaire**

***Dear student:***

This questionnaire is for the purpose of identifying the extent of perceived stress associated with the novel coronavirus (COVID-19) pandemic among students of the College of Medicine King Khalid University. Kindly answer clearly and objectively. Thank you very much.

***1- Personal data:***

- Age: …………….(years)

- Gender: Male / Female

- Academic level: ......................................................

- Grade Point Average (GPA):………………………………………..

-Family income: Sufficient and exceed/ Sufficient/ Insufficient

- Smoking status: Smoker/ Ex-smoker/ Non-smoker

***2- Perceived Stress Scale (PSS)***

The questions in this scale ask about your feelings and thoughts during the last month. In each case, you will be asked to indicate how often you felt or thought a certain way. Although some of the questions are similar, there are differences between them and you should treat each one as a separate question. The best approach is to answer fairly quickly. That is, don’t try to count up the number of times you felt a particular way; rather indicate the alternative that seems like a reasonable estimate.

| Question | Never | Almost never | Some-times | Fairly often | Very often |
| --- | --- | --- | --- | --- | --- |
| l. In the last month, how often have you been upset because of something that happened unexpectedly? |  |  |  |  |  |
| 2. In the last month, how often have you felt that you were unable to control the important things in your life? |  |  |  |  |  |
| 3. In the last month, how often have you felt nervous and stressed? |  |  |  |  |  |
| 4. In the last month, how often have you felt confident about your ability to handle your personal problems? |  |  |  |  |  |
| 5. In the last month, how often have you felt that things were going your way? |  |  |  |  |  |
| 6. In the last month, how often have you found that you could not cope with all the things that you had to do? |  |  |  |  |  |
| 7. In the last month, how often have you been able to control irritations in your life? |  |  |  |  |  |
| 8. In the last month, how often have you felt that you were on top of things? |  |  |  |  |  |
| 9. In the last month, how often have you been angered because of things that happened that were outside of your control? |  |  |  |  |  |
| 10. In the last month, how often have you felt difficulties were piling up so high that you could not overcome them? |  |  |  |  |  |

***3- COVID-19 related stressors:***

| Question | Never | Fairly often | Very often |
| --- | --- | --- | --- |
| 1. How worried are you about the effect of COVID-19 pandemic on your mental health? |  |  |  |
| 1. How worried are you about the effects of COVID-19 virus infection for your family and friends? |  |  |  |
| 1. How worried are you about the effects of COVID-19 virus infection for you personally? |  |  |  |
| 1. How worried are you about the effects of COVID-19 pandemic on academic flow and exams? |  |  |  |
| 1. How worried are you about the effects of COVID-19 pandemic control measures and lockdown? |  |  |  |
| 1. How do you think the numerous COVID-19 pandemic news causes stress to you? |  |  |  |
| 1. Is the afraid of stigma and isolation due to infection cause stress to you? |  |  |  |
| 1. Is the uncertainty about the pandemic future and its effects causes stress to you? |  |  |  |
