# Supplementary material for: Increased mental stress among undergraduate medical students in south-western Saudi Arabia during the COVID-19 pandemic
Source: PeerJ. 2022 Aug 15;10:e13900. doi: 10.7717/peerj.13900 (PMC9387517; doi:10.7717/peerj.13900)
Supplement: Supplemental Information 4 [file peerj-10-13900-s004.pdf]

COVID-19

عزيزتي الطالبة:
هذا الاستبيان يهدف للتعرف على الضغوط النفسيه المصاحبه لجائحة فيروس كورونا المستجد (COVID-19) بين طالبات كليه الطب - جامعه الملك خالد. و الغرض منه هو تدريب طالبات كليه الطب علي اجراء البحوث الصحية كجزء من مقرر طب المجتمع (طبع 431) في المستوي الثامن بالفصل الدراسي الأول للعام الجامعي 1442 . برجاء التكرم بالاجابة بوضوح وموضوعية و عدم كتابة ما يدل على شخصيتك او اسمك في هذا الاستبيان. شاكرين و مقدرين حسن تعاونكم معنا.
زميلاتكن طالبات المستوى الثامن - كلية الطب بمقرر طب المجتمع

* Required

1. العمر *

2. المستوى *

Mark only one oval.

- الثالث
- الرابع
- الخامس
- السادس
- السابع
- الثامن
- التاسع
- العاشر
- الحادي عشر
- الثاني عشر

3. المعدل التراكمي في السنة السابقة *

4. دخل الأسرة *

Mark only one oval.

- كافي و يفيض
- كافي بالكاد
- لا يكفي

5. التدخين *

Mark only one oval.

- تدخن
- لا تدخن

الأسئلة التاليه في هذا الاستبيان تتعلق بأحاسيسك وأفكارك خلال الشهر الماضي. ويطلب منكي في كل سؤال أن تبيني كم من الوقت أحسستي أو فكرتي بطريقة معينة. المرجو منكي أن تتعاملي مع كل سؤال على أساس أنه سؤال مستقل. والطريقة المثلى هي أن تجيبي على كل سؤال بسرعة، واختيار اجابة واحدة فقط

6. خلال الشهر الماضي كم من الوقت شعرتِي بالاضطراب نتيجة حصول حادثة غير متوقعة؟ *

Mark only one oval.

- لم يحدث أبدا
- تقريبا لم يحدث أبدا
- أحيانا
- في كثير من الأحيان ولكن الى حد ما
- كثيرا جدا

7. خلال الشهر الماضي كم من الوقت شعرتِي بعدم قدرتك على التحكم بالأمور المهمة في حياتك؟ *

Mark only one oval.

- لم يحدث أبدا
- تقريبا لم يحدث أبدا
- أحيانا
- في كثير من الأحيان ولكن الى حد ما
- كثيرا جدا

8. خلال الشهر الماضي كم من الوقت شعرتي بالتوتر و"الضغط النفسي"؟ *

Mark only one oval.

- لم يحدث أبدا
- تقريبا لم يحدث أبدا
- أحيانا
- في كثير من الأحيان ولكن الى حد ما
- كثيرا جدا

9. خلال الشهر الماضي كم من الوقت شعرتِي بالثقة حيال قدرتك على التعامل مع مشاكلك الشخصية؟ *

Mark only one oval.

- لم يحدث أبدا
- تقريبا لم يحدث أبدا
- أحيانا
- في كثير من الأحيان ولكن الى حد ما
- كثيرا جدا

10. * خلال الشهر الماضي كم من الوقت شعرتِي أن الأمور تجري حسب رغبتك؟

Mark only one oval.

- لم يحدث أبدا
- تقريبا لم يحدث أبدا
- أحيانا
- في كثير من الأحيان ولكن الى حد ما
- كثيرا جدا

11. * خلال الشهر الماضي كم من الوقت شعرتِي بعدم قدرتك على التكيف مع جميع الأمور التي عليك فعلها؟

Mark only one oval.

- لم يحدث أبدا
- تقريبا لم يحدث أبدا
- أحيانا
- في كثير من الأحيان ولكن الى حد ما
- كثيرا جدا

12. * خلال الشهر الماضي كم من الوقت شعرتِي انك قادرة على التحكم بمصادر الازعاج في حياتك؟

Mark only one oval.

- لم يحدث أبدا
- تقريبا لم يحدث أبدا
- أحيانا
- في كثير من الأحيان ولكن الى حد ما
- كثيرا جدا

13. خلال الشهر الماضي كم من الوقت شعرتِي انك تتحكمي بجميع الأمور؟ *

Mark only one oval.

- لم يحدث أبدا
- تقريبا لم يحدث أبدا
- أحيانا
- في كثير من الأحيان ولكن الى حد ما
- كثيرا جدا

14. خلال الشهر الماضي كم من الوقت شعرتِي بالغضب بسبب أمور خارجة عن سيطرتك؟ *

Mark only one oval.

- لم يحدث أبدا
- تقريبا لم يحدث أبدا
- أحيانا
- في كثير من الأحيان ولكن الى حد ما
- كثيرا جدا

15. خلال الشهر الماضي كم من الوقت شعرتِي ان المصاعب تتراكم لدرجة لا يمكنك التغلب عليها؟ *

Mark only one oval.

- لم يحدث أبدا
- تقريبا لم يحدث أبدا
- أحيانا
- في كثير من الأحيان ولكن الى حد ما
- كثيرا جدا

16. هل سببت لك جائحة كورونا الشعور بالتوتر؟ *

Mark only one oval.

- لم يحدث أبدا
- أحيانا
- كثيرا جدا

17. هل سبب لك الخوف من إصابة أحد أفراد الأسرة بفيروس كورونا المستجد الشعوربالتوتر؟ *

Mark only one oval.

- لم يحدث أبدا
- أحيانا
- كثيرا جدا

18. هل سبب لك الخوف من إصابتك بفيروس كورونا المستجد الشعور بالتوتر؟ *

Mark only one oval.

- لم يحدث أبدا
- أحيانا
- كثيرا جدا

19. هل سببت لك المشاكل المحتملة في الدراسة و الامتحانات نتيجة للجائحة الشعور بالتوتر ؟ *

Mark only one oval.

- لم يحدث أبدا
- أحيانا
- كثيرا جدا

20. هل سببت لك الاجراءات الاحترازية و الغلق المفروض من الدولة نتيجة للجائحة الشعور بالتوتر ؟ *

Mark only one oval.

- لم يحدث أبدا
- أحيانا
- كثيرا جدا

21. هل سبب لك الخوف من الموت بسبب الجائحة الشعور بالتوتر؟ *

Mark only one oval.

- لم يحدث أبدا
- أحيانا
- كثيرا جدا

22. هل سببت لك كثره الاخبار عن الجائحة الشعور بالتوتر ؟ *

Mark only one oval.

- لم يحدث أبدا
- أحيانا
- كثيرا جدا

23. هل سبب لك الخوف من العزلة أو الحرج في حالة معرفه اصابتك بفيروس كورونا المستجد الشعور بالتوتر ؟ *

Mark only one oval.

- لم يحدث أبدا
- أحيانا
- كثيرا جدا

24. هل سبب لك عدم وضوح المعلومات عن الجائحة الشعور بالتوتر ؟ *

Mark only one oval.

- لم يحدث أبدا
- أحيانا
- كثيرا جدا

25. هل سبب لك الاطلاع على أخبار الجائحة من مصادر غير موثوقه الشعور بالتوتر ؟ *

Mark only one oval.

- لم يحدث أبدا
- أحيانا
- كثيرا جدا

This content is neither created nor endorsed by Google.

Google Forms
